# Reduced cerebral vascular fractal dimension among asymptomatic individuals as a potential biomarker for cerebral small vessel disease

**DOI:** 10.1038/s41598-022-15710-9

**Published:** 2022-07-11

**Authors:** Niferiti Aminuddin, Anusha Achuthan, Nur Intan Raihana Ruhaiyem, Che Mohd Nasril Che Mohd Nassir, Nur Suhaila Idris, Muzaimi Mustapha

**Affiliations:** 1 Department of Neurosciences, School of Medical Sciences, Universiti Sains Malaysia, 16150 Kubang Kerian, Kelantan Malaysia; 2 Department of Basic Medical Sciences, Kulliyyah of Pharmacy, International Islamic University Malaysia, 25200 Kuantan, Pahang Malaysia; 3 School of Computer Sciences, Universiti Sains Malaysia, 11800 USM, Pulau Pinang Malaysia; 4 Hospital Universiti Sains Malaysia, Jalan Raja Perempuan Zainab II, 16150 Kubang Kerian, Kelantan Malaysia; 5 Department of Family Medicine, School of Medical Sciences, Universiti Sains Malaysia, 16150 Kubang Kerian, Kelantan Malaysia

**Keywords:** Neuroscience, Medical imaging

## Abstract

Cerebral small vessel disease is a neurological disease frequently found in the elderly and detected on neuroimaging, often as an incidental finding. White matter hyperintensity is one of the most commonly reported neuroimaging markers of CSVD and is linked with an increased risk of future stroke and vascular dementia. Recent attention has focused on the search of CSVD biomarkers. The objective of this study is to explore the potential of fractal dimension as a vascular neuroimaging marker in asymptomatic CSVD with low WMH burden. D_f_ is an index that measures the complexity of a self-similar and irregular structure such as circle of Willis and its tributaries. This exploratory cross-sectional study involved 22 neurologically asymptomatic adult subjects (42 ± 12 years old; 68% female) with low to moderate 10-year cardiovascular disease risk prediction score (QRISK2 score) who underwent magnetic resonance imaging/angiography (MRI/MRA) brain scan. Based on the MRI findings, subjects were divided into two groups: subjects with low WMH burden and no WMH burden, (WMH^+^; n = 8) and (WMH^−^; n = 14) respectively. Maximum intensity projection image was constructed from the 3D time-of-flight (TOF) MRA. The complexity of the CoW and its tributaries observed in the MIP image was characterised using D_f_. The D_f_ of the CoW and its tributaries, i.e., D_f_ (*w*) was significantly lower in the WMH^+^ group (1.5172 ± 0.0248) as compared to WMH^−^ (1.5653 ± 0.0304, *p* = 0.001). There was a significant inverse relationship between the QRISK2 risk score and D_f_ (*w*), (*r*_*s*_ = − .656, *p* = 0.001). D_f_ (*w*) is a promising, non-invasive vascular neuroimaging marker for asymptomatic CSVD with WMH. Further study with multi-centre and long-term follow-up is warranted to explore its potential as a biomarker in CSVD and correlation with clinical sequalae of CSVD.

## Introduction

Cerebral small vessel disease (CSVD) is a term used to describe spectrum of clinical and neuroimaging findings caused by pathological processes of various aetiologies affecting cerebral small vessels (i.e. small arteries, arterioles, venules, small veins, capillaries)^1^. CSVD can be classified into six (6) types based on the aetiology and pathologic aspects of the cerebral small vessel changes^1,2^. The aetiopathogenic classification of CSVD is manifested by diverse numbers of neuroimaging features such as WMH, lacunar infarction and cerebral microbleeds^3^. Given the insidious onset of the disease, CSVD could manifest without apparent neurological symptoms^4^. Typically, the symptoms and clinical sequalae of CSVD are recognized in the elderly subjects in its advanced stage^5^. Nonetheless, it is also important to note that younger CSVD patients could also present with symptoms at varying degrees, from subtle symptoms such as mood disorders^6^ to cognitive impairment^7^. Several studies involving community dwelling elderly and adults of younger age had estimated the prevalence of silent cerebral infarction to be in the range of 5 to 28%^8–10^. Importantly, CSVD had been held responsible for diverse number of clinical manifestations such as ischemic stroke, dementia, motor dysfunction, psychiatric disorders and epilepsy^1,11^. Of concern, approximately 25% of ischemic stroke and 45% of dementia have been contributed by CSVD^1,12,13^.

The silent nature and insidious manifestation of CSVD are among major challenges in our current understanding of its pathogenesis and development of -disease biomarkers. More recent attention has focused on the search of prospective biomarkers of CSVD by characterizing the cerebral parenchymal and cerebral vessels changes that occurred in CSVD^14^. In general, a disease biomarker may be used to aid diagnosis, stratify disease’s severity, predict the course of diseases and assess treatment response. In view of the CSVD manifestations complexity, collaborative approaches from diverse research fields such as medicine, mathematics and medical image analysis have been instigated to further expand current knowledge on CSVD’s biomarkers^2,15–17^. In this perspective, fractal analysis has gained recent attention among the researchers in biomedical sciences. Fractal analysis is a contemporary method that applied fractal geometry, a mathematical concept introduced by Benoit B. Mandelbrot that allowed us to describe and quantify the complexity of fractal structures in term of fractal dimension (D_f_)^18^. D_f_ is an index that measures the complexity of a self-similar and irregular structure such as blood vessels^18^. Reduced D_f_ is reflective of reduced complexity of the fractal structures of interest, and vice versa^18^. The potential of D_f_ to differentiate different disease states was previously reported for Alzheimer disease, autism spectrum, epilepsy, brain tumour and retinal tissue loss^19^.

Interestingly, retinal vascular D_f_ had been found to be lower in hereditary CSVD patients as compared to healthy controls^15^. Lower retinal vascular D_f_ had also been significantly associated with ischemic stroke^20^, cerebral microbleeds^21^ and deep WMH scores in a recent lacunar and mild cortical ischemic stroke patients^16^. In line with these findings, previous studies had demonstrated inverse association between retinal vascular D_f_ and major CSVD risk factors such as increased blood pressure^22–26^ and ageing^22,23,25,26^. Similar to the CSVD patients, the retinal vascular complexity was found to be significantly lower in the hypertensive^22^ and ageing subjects^27^ as compared to control subjects. Given the common embryological origin and vascular regulatory mechanisms possessed by the retinal and cerebral vascular structures, it has been suggested that retinal vascular may well reflect any corresponding cerebral vascular changes^28,29^. In a related perspective, the complexity of the circle of Willis and its tributaries confers a significant association between pathological changes in the larger-sized vessels and the markers of CSVD. A recent systematic review and meta-analysis concluded that carotid atherosclerosis was strongly associated with both silent brain infarction and cerebral WMH^30^. Similarly, a pooled analysis of cross-sectional studies consistently demonstrated association between greater arterial stiffness and the markers of CSVD^31^.

To our best knowledge, no previous study has explored fractal analysis approach in analysing the complexity of CoW and its tributaries in CSVD, herein referred to as D_f_ (*w*). Fundamentally, complexity changes of CoW and its tributaries would indicate aberrations in the cerebral vascular structure that could result in pathological cerebral parenchymal changes. Therefore, in this exploratory cross-sectional study, we attempted to estimate the complexity of the CoW and its tributaries using D_f_ and explored its potential as a novel vascular neuroimaging marker in asymptomatic CSVD subjects with low WMH burden. Based on the retinal vascular complexity studies done on CSVD and its related risk factors^15,16,20–26^, we postulated that D_f_ (*w*) manifested with a wide spectrum of CSVD may reflect the overall effect of vascular risk factors possessed by an individual on the cerebral blood vessels. This study aimed to discern the complexity of CoW and its tributaries as measured by D_f_ between the asymptomatic CSVD subjects with low WMH burden (WMH^+^) and subjects with no WMH burden (WMH).

## Materials and methods

### Study population

The data for this study were derived from a well-characterized cohort established from a recent study on CSVD based at a single centre, Hospital Universiti Sains Malaysia (HUSM), Kubang Kerian, Kelantan^32^. Approximately 96% of this region is predominated by Malay. Malaysian Chinese ethnicity accounts for about 3% of the local population^33^. This exploratory cross-sectional study was approved by the Human Research Ethics Committee Universiti Sains Malaysia (USM/JEPeM/15,030,096) and was conducted in accordance with the Declaration of Helsinki. Informed consent was obtained from all participants prior to the study. Participants of age between 25 to 75 years old with low to moderate QRISK2 score (i.e. QRISK2 score of < 20%^34^) were recruited from the Family Medicine Outpatient Clinic, HUSM. QRISK2 score is a risk assessment tool used to estimate the risk of developing cardiovascular diseases over the next 10 years. The QRISK2 score validation study included white, black African, black Caribbean, Chinese, South Asian, other Asian and other ethnicities^35^. The QRISK2 score was calculated using the QRISK2 web calculator (https://qrisk.org/) based on the demographic and clinical data of the study subjects which include the sex, age, ethnicity, smoking status, body mass index (BMI), systolic blood pressure, total cholesterol/high density lipoprotein (HDL) ratio, the presence of stage 4 or 5 chronic kidney disease, hypertension, diabetes, rheumatoid arthritis, atrial fibrillation and family history of premature coronary heart disease in the first degree relative (< 60 years old). All subjects had no known clinical manifestations of cerebrovascular disease and any history of cerebral vascular diseases. The risk assessment tool was used for the population of age between 25 and 84 years old. Hence, the lower limit of the participants age was set at 25 years old. The upper limit of the participants age was set based on the average life expectancy of the study population^36^.

**Figure 1:**
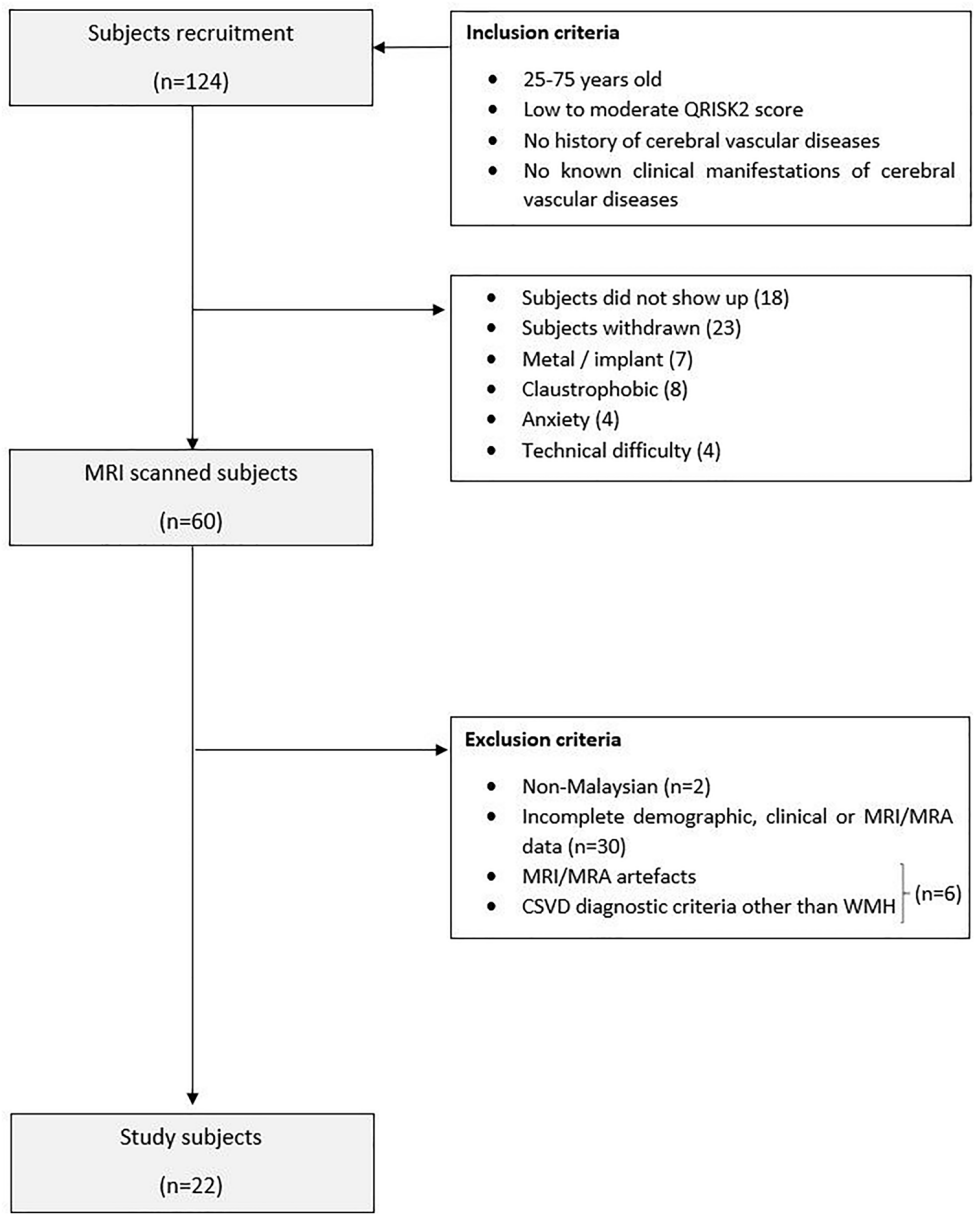
The study population’s inclusion and exclusion criteria.

Subjects of Malay and Chinese ethnicities (Malaysian) with normal MRI data or MRI data with the evidence of cerebral WMH of presumed vascular origin were included in the current study. Subjects with incomplete demographic, clinical, MRI/MRA data and/or MRI/MRA data with artefacts were excluded from this study. The inclusion and exclusion criteria of the study is illustrated in Fig. 1. The subjects were divided into two (2) groups, WMH^+^ (n = 8) and WMH^−^ (n = 14) based on the diagnostic evaluation done by the radiologist as per standards for reporting vascular changes on neuroimaging^37^. Visual ratings of the cerebral WMH was performed according to the Fazekas rating scale^38^.

The sample size for the current study was estimated prior to the study using G* power and satisfied statistical power of 80% at a 0.05 two-sided significance level. The sample size was estimated based on the data observed in a previous study related to retinal vascular complexity and CSVD^15^. The estimated total sample size for this study was 14 (7 in each group).

## Magnetic resonance imaging (MRI) acquisition protocol

Briefly, the MRI acquisition protocol included standard T1-weighted sequence, T2-weighted sequence, fluid- attenuated inversion recovery (FLAIR) sequence and 3DTOF MRA sequence. MRI acquisition was performed at 3.0 Tesla using a 32-channel head coil MRI scanner (Philips Achieva) with the *b*-value of 1000 s/mm^2^. The MRI acquisition parameters are as the followings; 3D-T1: echo time (TE) = 10 ms, repetition time (TR) = 678 ms, reconstruction matrix = 512 × 512 × 40, field of view (FOV) = 230 mm, voxel size = 0.45 mm × 0.45 mm, slice spacing = 0 mm, slice thickness = 2.5 mm, flip angle = 70°, and 180 contiguous sagittal slices orientation; T2: TE = 80 ms, TR = 3000 ms, reconstruction matrix = 512 × 512 × 24, FOV = 230 mm, voxel size = 0.45 × 0.45, slice spacing = 1.0 mm, slice thickness = 2.5 mm, and flip angle = 90°; 3D-fluid attenuated inversion recovery (FLAIR): TE = 125 ms, TR = 11,000 ms, TI = 2800 ms, reconstruction matrix = 512 × 512 × 24, FOV = 230 mm, voxel size = 0.45 mm × 0.45 mm, slice spacing = 0 mm, slice thickness = 2.5 mm, flip angle = 90°, and 170 contiguous sagittal slices orientation^32^. The image of the circle of Willis and its tributaries were captured using 3D-TOF MRA with the following parameters: TE = 3.5 ms, TR = 20 ms, reconstruction matrix = 720 × 720 × 200, FOV 200 mm, voxel size = 0.28 mm × 0.28 mm, slice spacing = 1.0 mm, slice thickness = 0.6 mm, flip angle = 20°.

### Image processing

#### MRA image pre-processing

The DICOM files were converted into NIfTI (Neuroimaging Informatics Technology Initiative) format. The 3D-TOF MRA images of each subject were spatially normalized into the standard International Consortium of Brain Mapping (ICBM) 152 template^39^ using FMRIB’s Linear Image Registration Tool, FLIRT (version 6.0.1) (www.fmrib.ox.ac.uk/fsl)^40^. Specifically, an affine 12 parameter model was applied to co-register the 3D-TOF MRA images to the T1-weighted average structural template image (ICBM 152, 0.5 × 0.5 × 0.5 mm)^41^. The co-registration step is essential to minimize the effects of variation in term of the head’s size and field of view positioning on the D_f_ (*w*).

#### Cerebral blood vessel segmentation, image binarization and skeletonization

The 2D-MIP MRA images of the whole brain were constructed from the co-registered 3D-TOF MRA images using MIP algorithm in Fiji software (version 1.53c)^42^. The 2D-MIP MRA images were stored in the form of Tagged Image File Format (TIFF). The 2D-MIP MRA images were semi-automatically segmented into two (2) segments, the cerebral blood vessels and background cerebral structures using fast random forest algorithm in Trainable Weka Segmentation Tools (version 3.2.33)^43^.

**Figure 2:**
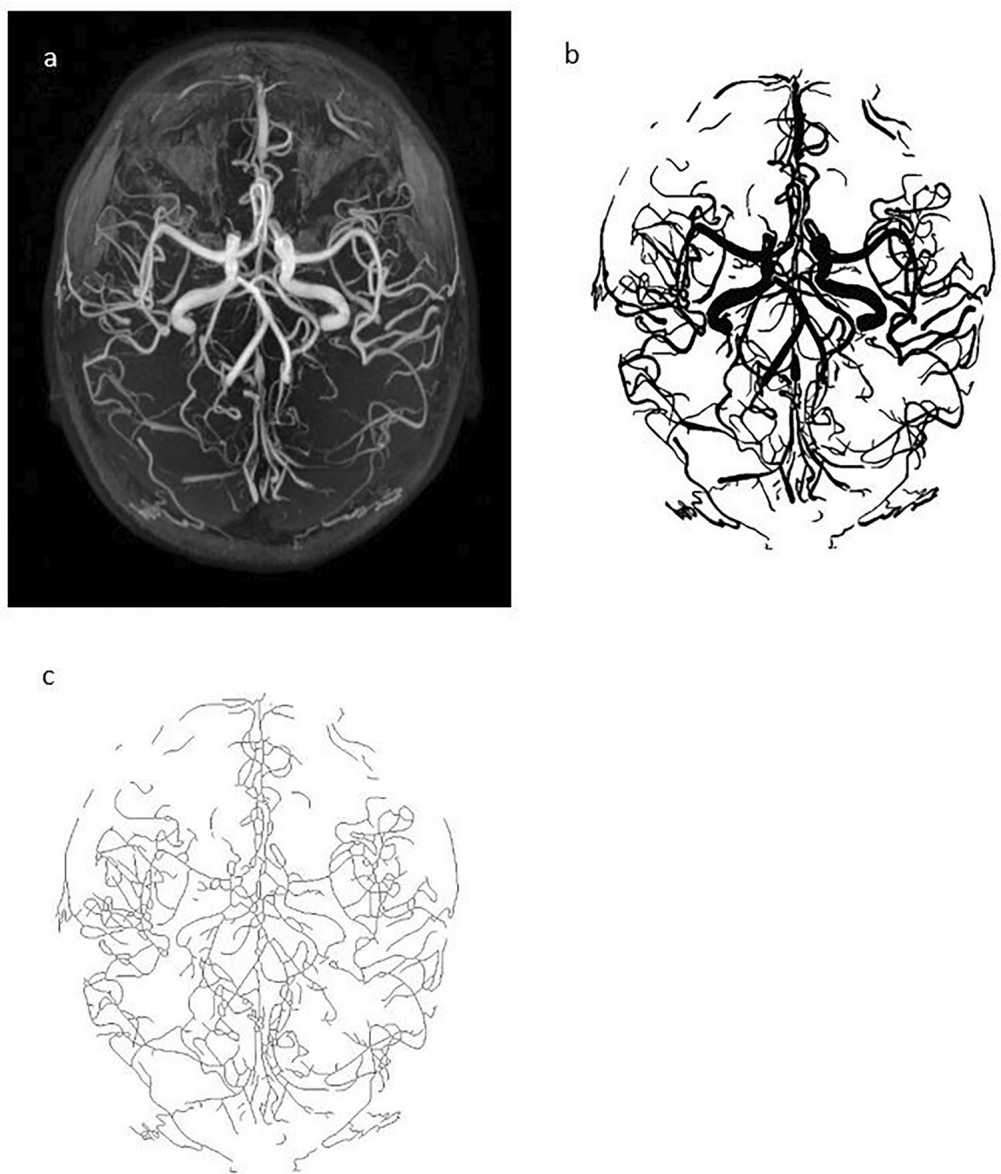
The figures illustrate the sequential image processing output in both WMH^+^ and WMH^−^ groups. (**a**) represents a 2D-MIP MRA image. (**b**) represents the corresponding binary image of the circle of Willis and its tributaries. (**c**) represents the corresponding skeletonized circle of Willis and its tributaries.

Afterwards, manual cerebral blood vessels segmentation was done using Fiji software (version 1.53c)^42^ on the output image until satisfactory cerebral blood vessel image was obtained. Artefacts were identified and erased. This step was performed by two (2) independent researchers with medical background who were trained by a radiologist. The researchers were blinded to the group allocation to reduce operator dependent bias. This procedure had resulted in two (2) different sets of cerebral blood vessel images. The segmented cerebral blood vessels images were saved as TIFF. Thereafter, the cerebral blood vessels images were converted into binary images and skeletonized (Fig. 2) using Fiji software (version 1.53c)^42^.The image binarization was done using default automatic thresholding function in Fiji Software (version 1.53c)^42^.

### Fractal analysis

Fractal analysis of the cerebral blood vessels of interest was performed using FracLac software (version 2.5) (http://rsb.info.nih.gov/ij/plugins/fraclac/FLHelp/Introduction.htm.)^44^. D_f_ (*w*) was calculated from the skeletonized cerebral blood vessel image using the box-counting method. The skeletonized image of the cerebral blood vessels was set as the foreground of the image, while the surroundings were set as the background of the image. The image was covered with a grid of linearly increasing box sizes until it reached 45% of the image size. The number of the non-empty boxes were counted at each scale ε. The steps were performed for 12 different grid positions. The average counts *N* of non-empty boxes at each scale ε was calculated. The average counts *N* was plotted against the scale ε in a double logarithmic plot. Linear regression of the plot was performed. Coefficient of determination *R*^2^ of 0.995 or higher was adopted as the goodness of fit measure of the regression line. The slope of the regression line was taken as the D_f_ (Eq. 1).

1 $$ {\text{D}}_{{\text{f}}} = {\text{ log}}N/{\text{ log }}\varepsilon $$

The D_f_ (*w*) obtained from the dataset of the two (2) independent researchers were averaged.

### Statistical analysis

All statistical analyses were performed with the SPSS statistics for Windows, version 23.0^45^. Fisher’s exact test was used to test the differences in proportion. Intraclass correlation coefficient (ICC) was used to analyse inter-rater reliability of the D_f_ measurement using our method. The ICC estimate, and its 95% confidence interval was calculated based on absolute agreement and 2-way mixed-effects model. Further statistical analysis was conducted using the averaged D_f_ (*w*). The statistical differences in D_f_ (*w*) between the asymptomatic WMH^+^ and WMH^-^ groups were analysed using independent T test (2-tailed) as the data was normally distributed. The box and whisker plot was generated using ggplot2 package in R programming language for statistical computing^46,47^.

The relationship between a well-established 10-year cardiovascular disease risk prediction score (i.e. QRISK2 score) and D_f_ (*w*) were analysed using Spearman correlation test as the QRISK2score data was not normally distributed. The normality of the data was assessed using Shapiro–Wilk's test. Values reported were expressed in mean ± standard deviation. The significance level was set at *p* < 0.05 (2-tailed).

## Results

In total, 124 subjects were initially recruited with predominant female subjects (68%). Of these, 60 subjects met all the inclusion criteria and underwent MRI whole brain examination. For the purpose of the current study, datasets from 22 subjects were included after considering the exclusion criteria (Fig. 1). Table 1 (a) summarizes the demographics and clinical variables of the study participants. BMI was reported based on the World Health Organization classification^48^. All subjects in the WMH^+^ group had Fazekas grade 1. Meanwhile, all subjects in the WMH^-^ group had Fazekas grade 0. Table 1 (b) summarizes the Fazekas grade, mean age, BMI, systolic blood pressure, total cholesterol/HDL ratio and QRISK2 score of the WMH^+^ and WMH^-^ groups.

**Table 1:** (a) Demographics and clinical variables of the study participants. (b) The Fazekas grade (c) The mean age, BMI, systolic blood pressure, total cholesterol/HDL ratio and QRISK2 score of the WMH^+^ and WMH^−^ groups.

(a) Demographic and clinical variables		WMH^+^ n (%)	WMH^−^ n (%)	Total n (%)
Sex	Male Female	2 (9.09) 6 (27.27)	5 (22.73) 9 (40.91)	7 (31.82) 15 (68.18)
Ethnicity	Malay Chinese	8 (36.36) 0 (0.00)	11 (50.00) 3 (13.64)	19 (86.36) 3 (13.64)
Smoking status	Non-smoker Ex-smoker Light smoker	7 (31.82) 1 (4.55) 0 (0.00)	12 (54.55) 1 (4.55) 1 (4.55)	19 (86.36) 2 (9.09) 1 (4.55)
Hypertension	Yes No	4 (18.18) 4 (18.18)	1 (4.55) 13 (59.09)	5 (22.73) 17 (77.27)
BMI	Underweight Normal Pre-obesity Obesity class I	0 (0.00) 2 (9.09) 5 (22.73) 1 (4.55)	2 (9.09) 9 (40.91) 3 (13.64) 0 (4.55)	2 (9.09) 11 (50.00) 8 (36.36) 1 (4.55)
Hyperlipidaemia	Yes No	0 (0.00) 8 (36.36)	0 (0.00) 14 (63.64)	0 (0.00) 22 (100.00)
Diabetes	Yes No	0 (0.00) 8 (36.36)	0 (0.00) 14 (63.64)	0 (0.00) 22 (100.00)
Chronic kidney disease (stage 4 or 5)	Yes No	0 (0.00) 8 (36.36)	0 (0.00) 14 (63.64)	0 (0.00) 22 (100.00)
Atrial fibrillation	Yes No	0 (0.00) 8 (36.36)	0 (0.00) 14 (63.64)	0 (0.00) 22 (100.00)
Rheumatoid arthritis	Yes No	1 (4.55) 7 (31.82)	0 (0.00) 14 (63.64)	1 (4.55) 21 (95.45)
Family history of premature coronary heart disease in the first degree relative (< 60 years old)	Yes No	3 (13.64) 5 (22.73)	2 (9.09) 12 (54.55)	5 (22.73) 17 (77.27)

		WMH^+^	WMH^-^	Total
(b) Fazekas grade		n (%)	n (%)	–
	0	–	14 (100%)	–
	1	8 (100%)	–	–

		WMH^+^	WMH^-^	Total
(c) Clinical variables		Mean ± SD	Mean ± SD	Mean ± SD
Age (years)		47 ± 13	38 ± 11	42 ± 12
BMI (kg/m^2^)		26.2 ± 3.2	22.6 ± 3.1	23.9 ± 3.6
Systolic blood pressure (mmHg)		139.63 ± 16.21	123.14 ± 11.73	129.14 ± 15.45
Total cholesterol/HDL ratio		3.26 ± 0.49	3.82 ± 0.63	3.62 ± 0.63
QRISK2 score		5.84 ± 5.83	1.55 ± 1.79	3.11 ± 4.22

WMH^+^, asymptomatic CSVD subjects with low white matter hyperintensities burden; WMH^−^, subjects with no white matter hyperintensities burden ; BMI, body mass index; HDL, high density lipoprotein; SD, standard deviation; n, sample size; Light smoker, smoking less than 10 cigarettes per day ; Underweight, BMI of below 18.5 kg/m^2^; normal, BMI of 18.5–24.9 kg/m^2^; pre-obesity, BMI of 25.0– 29.9 kg/m^2^; obesity class I, BMI of 30.0–34.9 kg/m^2^; Hypertension, systolic blood pressure of ≥ 140 mmHg^49^.

Altogether, 22 subjects were divided into two (2) groups; WMH^+^ (n = 8) and WMH^−^ (n = 14). The age of the study subjects ranged between 25 to 62 years old. The mean age of the study population was 42 ± 12 years old. There were seven (7) male and fifteen (15) female subjects. There were no significant differences in term of the sex proportion between the two (2) groups as indicated by Fisher’s exact test (*p* > 0.05).

**Figure 3:**
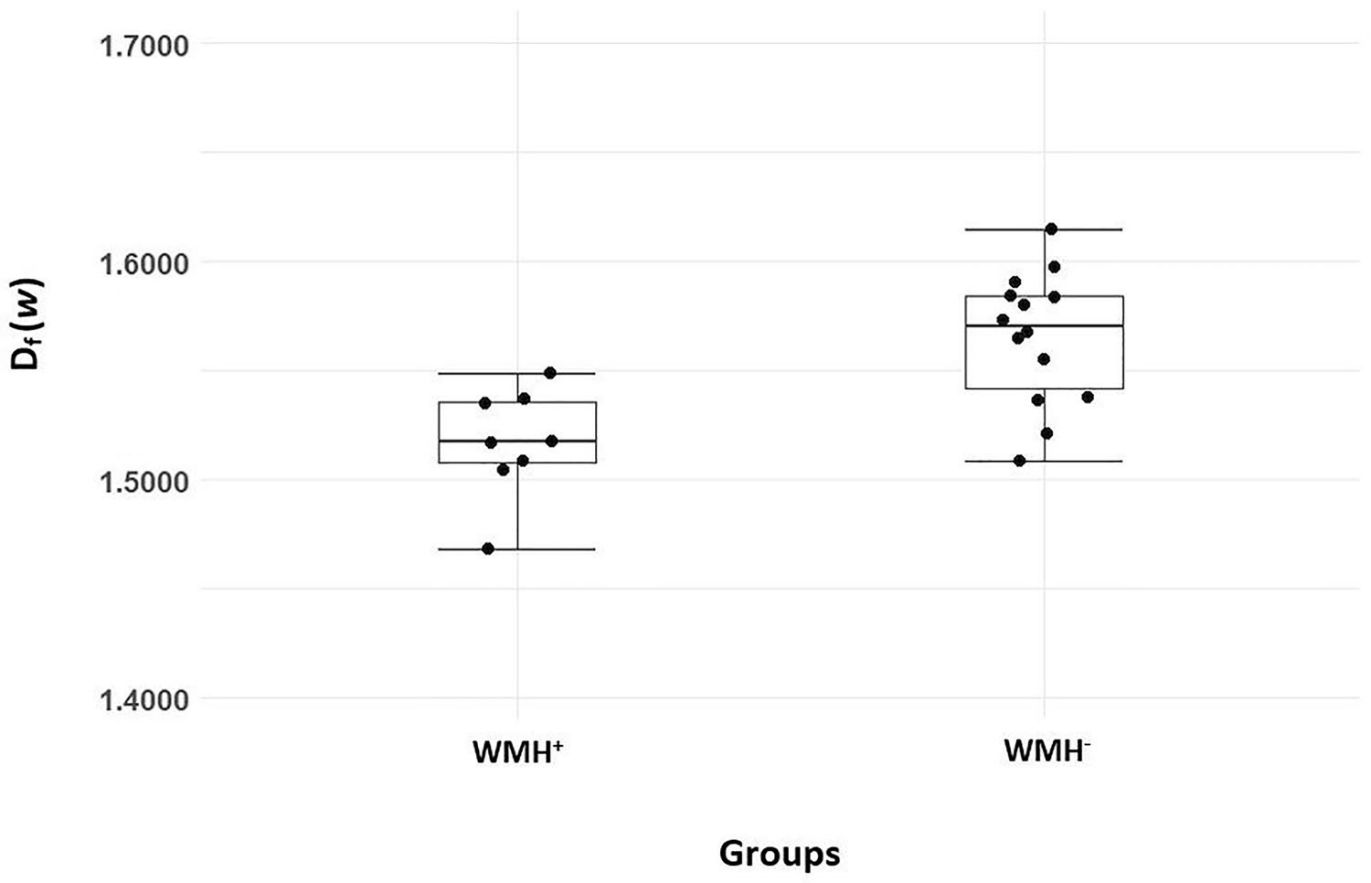
The distribution of the D_f_ (*w*) in WMH^+^ and WMH^-^ groups.

**Figure 4:**
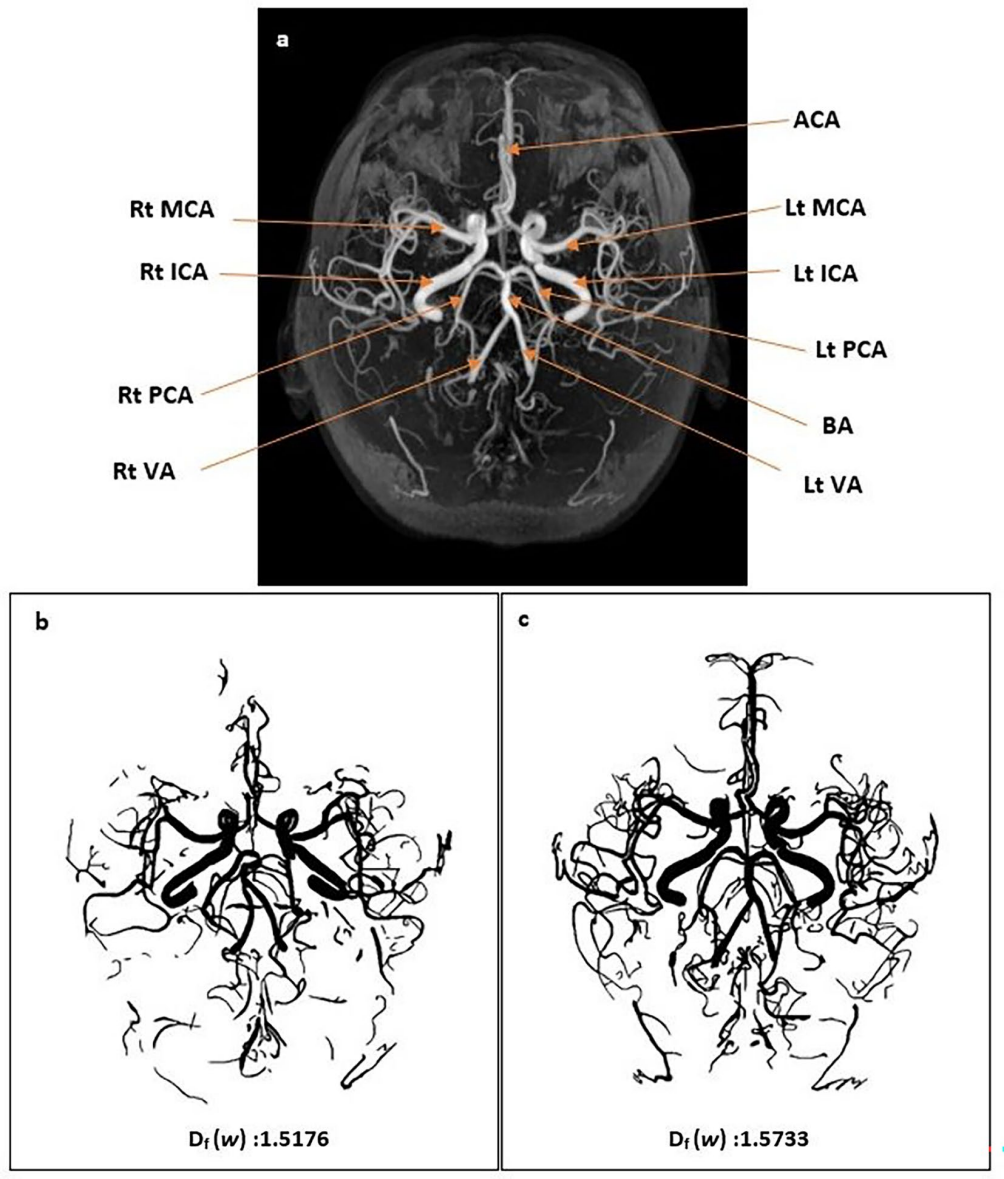
(**a**) Illustrates the circle of Willis and its tributaries. Rt: right; Lt: Left; ICA: internal carotid artery; MCA: middle cerebral artery; ACA: anterior cerebral artery; PCA: posterior cerebral artery; BA: basilar artery; VA: vertebral artery. Figure (**b**) and (**c**) illustrates the circle of Willis and its tributaries and D_f_ (*w*) of the subjects that presented with low WMH burden and no WMH burden respectively.

The inter-rater reliability estimates for D_f_ was excellent with ICC (95% confidence interval) 0.99 (0.99–1.00). The mean D_f_ (*w*) was lower in the WMH^+^ group (1.5172 ± 0.0248) as compared to WMH^−^ group (1.5653 ± 0.0304), a statistically significant difference of 0.0481 (95% CI, 0.0229 to 0.0733), *t* (17.282) = 4.018, *p* = 0.001 (2-tailed). The distribution of the D_f_ (*w*) for both groups are shown in Fig. 3. The image of CoW and its tributaries and D_f_ (*w*) of subjects that presented with low WMH burden and no WMH burden respectively, as illustrated in Fig. 4.

**Figure 5:**
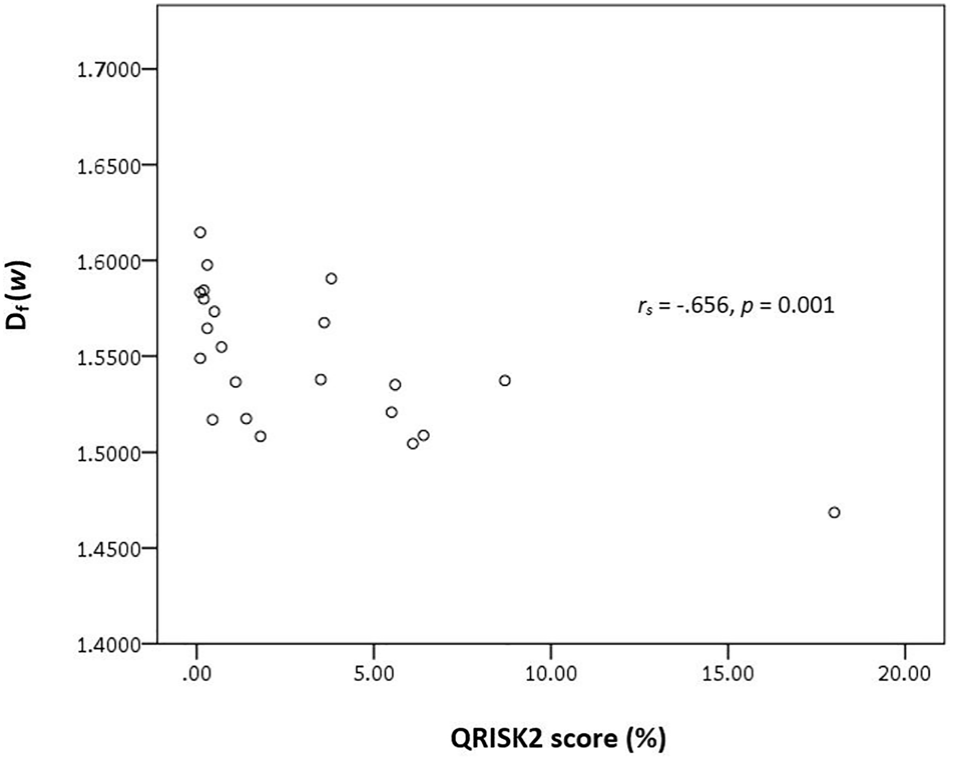
The scatterplot illustrates the relationship between QRISK2 score and D_f_ (*w*).

An outlier was noted in the preliminary analysis of Spearman correlation test. The Spearman correlation test demonstrated a significant and strong, negative correlation between the QRISK2 score and D_f_ (*w*) (n = 22; *r*_*s*_ = − 0.656, *p* = 0.001). The relationship between QRISK2 score and D_f_ (*w*) is illustrated in Fig. 5. A repeated test without the outlier had revealed no appreciable difference in term of the result (n = 21; *r*_*s*_ = − 0.604, *p* = 0.004).

## Discussion

In previous studies, neuroimaging techniques have been used to assess the cerebral blood flow, cerebral vascular reactivity and blood brain barrier function in CSVD^14,50^. To our knowledge, this is the first study that had applied fractal analysis as an approach to characterize the complexity of CoW and its tributaries in asymptomatic CSVD subjects with low WMH burden. Our study had demonstrated significantly lower D_f_ (*w*) in asymptomatic CSVD subjects with low WMH burden (1.5172 ± 0.0248) in comparison to control subjects (1.5653 ± 0.0304), *p* = 0.001. Under the 3D-TOF MRA (3-Tesla) examination, our study revealed reduced complexity of CoW and its tributaries in the asymptomatic CSVD subjects with low WMH burden. In support of our finding, past research had suggested the role of retinal vascular and cerebral white matter D_f_ as one of the potential CSVD’s biomarkers^15–17^.

Importantly, the study findings lend support to previous research that argued that larger-sized blood vessels might be involved in the pathogenesis of CSVD^31,51,52^. It is still not yet understood whether the cerebral small vessel structural changes preceded the larger-sized blood vessels structural changes or vice versa^53^. An increase in the stiffness of larger arterial vessels is thought to inflict damage to the vulnerable cerebral small vessels from the heightened flow load and pulsatile pressure^31,53–55^. Whereas, in small vessel diseases, the vascular changes lead to haemodynamic alteration that cause vicious cycle of small and large vessels injuries and damage^51^. Nonetheless, these findings suggest that CSVD patients might benefit from targeted assessments of the varied-sized vascular tree involvement as a part of the disease natural history.

It is plausible that reduced complexity of CoW and its tributaries could be observed in the asymptomatic subjects with CSVD involved both small and larger-sized blood vessels as both are structurally and functionally connected^56^. In fact, the presence of reduced retinal vascular complexity in the CSVD patients indirectly suggest that similar changes might have occurred in the cerebral small vessels of CSVD patients^15,16,20,21^. In support of this notion, a recent study had revealed positive association between cerebral blood flow and retinal vascular D_f_ in a cohort of healthy elderly subjects^57^. Besides, an experimental animal study had demonstrated that cerebral microvascular rarefactions precede the evidence of decreased cerebral blood flow and subsequent white matter lesion in CSVD^58^. A more direct assessment of the cerebral small vessels would require an examination under advanced magnetic imaging at ultra-high field (7-Tesla). However, 7-Tesla MRI is not widely available in both clinical and research setting.

Interestingly, our study had revealed that D_f_ (*w*) could detect early and subtle changes in term of the complexity of CoW and its tributaries in the asymptomatic CSVD with low WMH burden subjects despite having low 10-year predictive cardiovascular risk score. In our current study, the evidence of reduced D_f_ (*w*) and cerebral WMH in a relatively young population is rather concerning. The age of the subjects in this study ranged between 25 and 62 years old. In accordance with the current knowledge, the mean age of the WMH^+^ (47 ± 13 years old) in this study was higher than the WMH^−^ group (38 ± 11 years old). Similarly, we had also noted that the mean BMI and systolic blood pressure was higher in the WMH^+^ (26.2 ± 3.2 kg/m^2^; 139.63 ± 16.21 mmHg) than the WMH^−^ group (22.6 ± 3.1 kg/m^2^; 123.14 ± 11.73 mmHg). In contrary, the mean total cholesterol/HDL ratio was found to be higher in the WMH^-^ (3.82 ± 0.63) than the WMH^+^ group (3.26 ± 0.49). Nonetheless, the mean total cholesterol/HDL ratio were within normal range for both groups^59^. Up until now, the association between lipid and CSVD remains inconclusive despite the recognised hyperlipidaemia as one of the CSVD risk factors^13,60^. Conversely, several studies had shown that hyperlipidaemia was inversely associated with WMH severity^61–63^. For instance, previous studies had demonstrated that hyperlipidaemia was inversely associated with WMH severity in acute ischemia stroke^62^. Similarly, Ke et al., 2018 had demonstrated that hypertriglyceridemia is inversely associated with WMH severity^61^. Intriguingly, hypercholesterolemia has been reported to lower risk of CSVD. It was suggested that elevated cholesterol might be protective against cerebral injury as it plays an important role in the development and maintenance of new synapses^62,63^. As CSVD is commonly diagnosed in the ageing population, the evidence suggests that the early stage of CSVD might had begun much earlier and took years to progress and became symptomatic. In line with the current study, previous studies also supported the potential of neuroimaging markers of CSVD in the younger adult population and that CSVD severity worsened in the older adults^7,64^. It was also noted that the severity of the disease increases in the older population^7,64^. This signifies that there is a large window of opportunity for earlier intervention if the susceptible subjects could be detected at an earlier stage of the disease. Besides, our results had also demonstrated a significant negative correlation between the QRISK2 score, an established 10-year predictive cardiovascular risk prediction score and D_f_ (*w*) (*r*_*s*_ = − 0.656, *p* = 0.001). In the validation study of QRISK2 score, the term cardiovascular disease includes coronary heart disease, stroke and transient ischemic attack hence this indicate that QRISK2 score is non-specific for cerebral vascular diseases^35^. Distinct from the QRISK2 score, D_f_ (*w*) offers a quantitative measure of the structural changes observed on CoW and its tributaries. Noteworthy, CoW and its tributaries are directly involved in perfusing the cerebral parenchyma. D_f_ (*w*) is complementary to the cerebral small vessel complexity measures offered by retinal vascular D_f_. Indeed, cerebral WMH is a well-established diagnostic marker of CSVD that reflects the integrity of cerebral parenchyma rather than the affected vascular structure itself. Therefore, D_f_ (*w*) could provide additional information regarding the state of the underlying vascular structural changes. In the context of CSVD biomarker, D_f_ (*w*) might be of use in combination with other imaging and molecular biomarkers of CSVD. For instance, retinal vascular D_f_ and other biomarkers of CSVD could be used as a screening modality to determine the candidate that might benefit from a whole brain MRI/MRA examination. Further studies, including the potential of D_f_ (*w*) in stratification of disease severity in symptomatic CSVD patients are warranted, and may help to establish its prognostic values and potential to evaluate treatment response. Notwithstanding, long-term prospective studies are warranted to assess the sensitivity and specificity of fractal analysis approach in characterizing the complexity of CoW and its tributaries in CSVD subjects.

One of the limitations of this study is a relatively small sample size used in the study. Therefore, a larger-scaled study is required to confirm our findings. However, a larger-scaled study would require a more efficient cerebral blood vessels segmentation method that is less time-consuming, such as a fully automated segmentation method. To the best of our knowledge, an established software that enable us to efficiently and fully automatically segment the cerebral blood vessels observed on the MRA image is not yet available. The major challenges in fully automated segmentation method is to capture all cerebral blood vessels on the MRA image in view of the structural variation in the normal and pathological vessels, the tortuosity and overlapping of the vascular structure, and the similarities in term of the signal intensity of the vascular structure to other brain structures. More recent attention has focused on exploration of a more advanced machine learning approach in vascular segmentation technique^65^. It is hoped that future advancement in the cerebral vascular segmentation technique would make a larger-scaled study more feasible. Besides, there are several other approaches that might be more optimum for registration of 3D-TOF MRA images to the T1-weighted average structural template image^66,67^. More studies are required to explore the best registration method that would be suitable for D_f_ estimation of the CoW and its tributaries. Furthermore, this study only focused on the cerebral WMH as the diagnostic neuroimaging feature of CSVD. Noteworthy, cerebral parenchymal changes such as cerebral microinfarct and perivascular spaces might be undetectable under lower-field (less than 7-Tesla) MRI examination^4,68^. Nonetheless, other studies are needed to analyse the complexity of CoW and its tributaries in CSVD patients with other diagnostic neuroimaging features of CSVD. The strengths of this study include the non-invasive and radiation-free nature of the 3D-TOF MRA. Importantly, the 3D-TOF MRA (3-Tesla) is accessible in both research field and standard clinical practice.

In summary, our study had revealed that asymptomatic CSVD subjects (from the incidental MRI finding of the presences of low WMH burden) had significantly lower D_f_ (*w*) in comparison to the control subjects. Significant inverse correlation was also found between the QRISK2 score and D_f_ (*w*). These findings suggest that D_f_ (*w*) may serve as a promising imaging biomarker for asymptomatic CSVD subjects from the presence of low WMH burden. Future studies on broader population are required to explore its potential in CSVD and in predicting its clinical sequalae such as cognitive impairment and stroke.

## Data Availability

The datasets generated are not publicly available due to privacy or ethical restrictions but are available from the corresponding author on reasonable request with permission from the Director, Hospital Universiti Sains Malaysia (USM).
